# A Practice-Distributed Thunder-Localization System with Crowd-Sourced Smart IoT Devices †

**DOI:** 10.3390/s23094186

**Published:** 2023-04-22

**Authors:** Bingxian Lu, Ruochen Wang, Zhenquan Qin, Lei Wang

**Affiliations:** School of Software, Dalian University of Technology, Dalian 116000, China; bingxian.lu@dlut.edu.cn (B.L.);

**Keywords:** acoustic source localization, crowdsensing, thunder localization, IoT

## Abstract

Lightning localization is of great significance to weather forecasting, forest fire prevention, aviation, military, and other aspects. Traditional lightning localization requires the deployment of base stations and expensive measurement equipment. With the development of IoT technology and the continuous expansion of application scenarios, IoT devices can be interconnected through sensors and other technical means to ultimately achieve the goal of automatic intelligent computing. Therefore, this paper proposes a low-cost distributed thunder-localization system based on IoT smart devices, namely ThunderLoc. The main idea of ThunderLoc is to collect dual-microphone data from IoT smart devices, such as smartphones or smart speakers, through crowdsourcing, turning the localization problem into a search problem in Hamming space. We studied the dual microphones integrated with smartphones and used the sign of Time Difference Of Arrival (TDOA) as measurement information. Through a simple generalized cross-correlation method, the TDOA of thunderclaps on the same smartphone can be estimated. After quantifying the TDOA measurement from the smartphone node, thunder localization was performed by minimizing the Hamming distance between the binary sequence and the binary vector measured in a database. The ThunderLoc system was evaluated through extensive simulations and experiments (a testbed with 30 smartphone nodes). The extensive experimental results demonstrate that ThunderLoc outperforms the main existing schemes in terms of effectively locating position and good robustness.

## 1. Introduction

The lightning-localization system is of great significance in various applications, such as weather forecasting, forest fire prevention, aviation, the military, power system failure risk analysis, and electromagnetic interference research [1]. Lightning activity generally occurs in strong convective weather. Strong lightning activities may cause forest fires or cause injury to people and cause casualties, and may also cause adverse effects on electronic communication equipment [2,3]. The development of modern lightning-localization systems can monitor lightning activity in a larger area in real-time. Locating lightning activities in the area can effectively reduce the casualties and property losses caused by lightning and minimize the adverse effects of lightning activities on electronic communication equipment.

With the development of IoT, requirements such as low energy consumption, transmission range, and cost-effectiveness are crucial for IoT applications. Traditional lightning-localization systems based on electromagnetic radiation require the deployment of base stations and expensive special equipment [4], making them unable to achieve low-cost goals. In recent years, centralized microphone array-based solutions for sound source localization have used multiple synchronized microphones to simultaneously acquire sound signals. For large-scale applications, this method has some limitations with the distance between the microphones and the sensing range.

A Wireless Sensor Network (WSN) is a wireless network composed of a series of sensor nodes that communicate through certain protocols and methods. It aims to use sensors to detect the target area and transmit the collected data to the network owner for specific use. Usually, it is necessary to maintain the node, dynamically adjust the network topology, and design algorithms to reduce the data transmission volume.

With the developments in mobile computing and communication technology, most smart devices are equipped with one or more microphones [5,6,7]. The development of Internet of Things (IoT) technology has provided various IoT devices with capabilities such as wireless sensing communication and data transmission, which can interconnect multiple users while achieving IoT robustness [8]. Due to the latest developments in IoT smart devices with rapid deployment capabilities, thunder localization via Wireless Acoustic Sensor Networks (WASN) based on IoT devices has become feasible.

The thunder localization via WASN used IoT device sensors to collect sound signals, transfer valuable data information to the cloud platform through the network, and use a series of localization algorithms in the cloud platform to calculate the location of the sound source. A single microphone can be used as a sensor node to collect sound signals. Because a single microphone has the shortcomings of being susceptible to noise interference and having a small sensing distance, people have begun to use and study microphone arrays. Usually, a microphone array has multiple synchronized microphones, which can collect multiple simultaneous channels. The sound signal has strong anti-noise interference ability, and the perception distance has also been improved. At present, there are still some problems with the sound source localization based on the microphone array in the wireless sensor network, and researchers need to further study and explore how to achieve finer clock synchronization between the microphone arrays, how to obtain more accurate measurement data values, and how to improve positioning range and accuracy.

Through wireless communication, the limitation of the size of the microphone array disappears, and WASN under IoT can cover a larger area.

**Figure 1:**
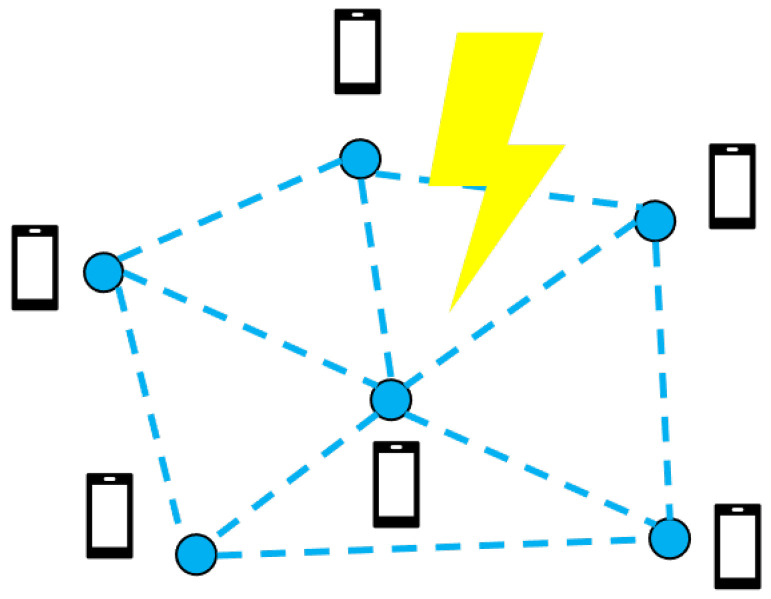
Thunder Localization.

Inspired by works [9,10], we propose a new method for thunder localization using dual-microphone IoT smart devices. For commercial dual-microphone smartphones in IoT devices, the binary left/right data of the dual-microphones of each smartphone can be effectively collected and used to estimate the location of thunder. As shown in Figure 1, the proposed system only relies on the cooperation of participating users to realize thunder localization.

The ThunderLoc project implemented a prototype system using different types of Android phones. It demonstrates the proposed potential solution by virtually researching the thunder inspection certificate our approach. ThunderLoc makes full use of the sensor data on the smartphone, including the sound, position, and direction provided by the microphone, GPS, accelerometer, and magnetometer. The key idea of ThunderLoc is to divide the 2D localization space into different areas by dividing the vertical bisector of the line connecting the dual microphones in each smartphone. Each different area formed in this way can be uniquely identified using a binary sequence. We first construct a binary sequence table and map all these feasible binary sequences to the corresponding area by using the location and direction information of the smartphone node. The smartphone node determines the measured binary sequence according to the TDOA symbol between the two microphones of each smartphone node. By searching the binary sequence table to determine the closest feasible sequence to the measurement sequence, the position of the sound source can be estimated. As far as we know, this is the first time in the open literature that crowdsourcing technology based on smartphones has been applied to thunder localization. The extensive experimental results demonstrate that ThunderLoc outperforms the main existing schemes in terms of good robustness and the effective location of the position. Our contributions are as follows:

- We designed and implemented ThunderLoc, a thunder-localization system based on crowdsourcing. More than 1000 users of our university downloaded our ThunderLoc app for the Android platform to monitor thunder near the campus. As far as we know, ThunderLoc is the first thunder-localization service based on crowdsourcing.
- There is no need for high-precision time synchronization between smartphones. ThunderLoc relies on measuring the TDOA symbol between the two synchronized microphones of each smartphone, thereby reducing the time synchronization requirements.
- The localization scheme has a high fault tolerance. The left/right binary data and the novel localization method make the localization system more robust to the position error, direction error, and measurement error of the smartphone node.
- There is low communication overhead and computational complexity. Each smartphone in the sensor network transmits 1 bit of measurement information, and a simple cross-correlation algorithm is sufficient to estimate the binary left/right data.

## 2. Related Work

Lightning is a discharge phenomenon with a large current, high voltage, and strong electromagnetic radiation in the atmosphere, which often causes serious casualties and economic losses. Therefore, fast and accurate lightning locating is very necessary. Thunder-localization systems can use infrasonic signals, supersonic signals, and audible signals as measurement data. Most of the existing thunder-localization systems using audible sound data are based on a centralized architecture.

Wood, et al. [11] used the large spacing between the two receivers; ambiguity sometimes causes the signal detected at one site to pair with a matching signal detected at the other. In these cases, the sferic (lightning discharge) of two receivers is used to generate an electromagnetic pulse and propagate through the waveguide formed by the earth and the ionosphere. The best estimation of a lightning-discharge location is obtained by comparing the difference in arrival azimuth and arrival time between receivers. Shanqiang, et al. [12] proposed a method based on endpoint detection and noise estimation to identify lightning signals. Two sound signals are selected to test the endpoint-detection method. The noise estimation algorithm is used to test the highly non-stationary environment to obtain the thunder signal, the exact start time, and the end time. Qiu, et al. [13] used a 3-element array with a 100 k sample rate and 12.5 m baseline to estimate the location of thunderclaps. Kadlec, et al. [14] used a fast lightning location technique, which is completed completely in the time domain. The overhead transmission line is located above the ideal ground, and its voltage response is calculated analytically, which speeds up the optimization of the global heuristic algorithm. Jin, et al. [10] proposed a new method for thunder location using a commercial microphone smartphone. The binary data of the microphone in the mobile phone is used to search the binary sequence table to determine the closest feasible sequence to the measurement sequence to estimate the location of thunder. Li, et al. [15] used a key parameter for evaluating the pros and cons of a localization system, and the numerical verification of position errors and the reasonable layout of stations are essential to improving localization accuracy.

The distribution of lightning sources in the lightning channel is studied using the radio frequency pulse positions drawn by the Lightning Mapping Array (LMA) [16]. The least squares method is used to retrieve the fitting channel acoustic energy radiation and broadband acoustic records, and the microphone is deployed near the lightning. We reconstruct the lightning channel geometry of clouds and ground flashes by locating the temporal and spatial changes of the lightning source [17] and use the thunder channel reconstruction model of three, four, and five microphone systems to calculate the error accuracy. Wu, et al. [18] used a wireless sensor network with random topology, which is used to solve the problem of target location. Through special decomposition technology to exchange local information with neighboring anchor nodes, the centralized Gauss–Newton method is modified into a distributed solution, which has the ability to resist environmental noise while ensuring convergence. In this paper, an adaptive penalty function semidefinite programming (apf-sdp) algorithm is proposed, which can avoid excessive penalty by adaptively selecting the penalty coefficient. No matter whether the noise level is large or not, the proposed intelligent apf-sdp algorithm is superior in location and speed estimation. Zhou, et al. [19] used a two-stage RBL method, which uses the RBL framework of a single base station to fuse the AOA measurements and the topology information of RBL more effectively. Finally, the method is evaluated as having a significantly faster speed.

Compared with the traditional localization using human ears [20], mobile phone voice localization has higher resolution and accuracy. In addition, compared with using images of lightning [21] to locate lightning, sound has higher robustness in locating thunder.

Sound source localization algorithms based on TOA and TDOA have provided rich theoretical support for sound source localization research. According to a series of discussions and methods developed using TOA, the methods have made outstanding contributions in terms of noise reduction, improvement of localization accuracy, and robustness. Compared with the TOA-based localization algorithm, the TDOA-based sound source localization uses the time difference between the sound source’s arrival at two nodes instead of the absolute time of arrival value, which is higher in accuracy than the TOA-based localization algorithm. This article is based on TDOA for ranging and localization.

Liu, et al. [22] achieved keystroke recognition by discriminating keystrokes based on TDOA of the keystroke sound at the dual-microphone of the off-the-shelf smartphone. Veibäck, et al. [23] proposed a method of sound source localization and reconstruction using a wearable microphone array and inertial sensors.Different from these range-based methods, our proposed ThunderLoc is a range-free method, which allows the transmission of data from dual-microphone smartphones in IoT devices to the cloud platform with a crowdsensing mechanism and, thus, can achieve robust localization performance.

The most related work to our ThunderLoc project is Lightning@SG, an app developed by the National Environment Agency (NEA) in Singapore [24]. By utilizing the four sensors located in different cities, Lightning@SG monitors the lightning area based on the current location of the user and pushes notifications of the lightning situation.

With the introduction and development of the IoT, large-scale applications and interconnections between devices form the IoT sensing network. It can provide a robust operating environment for localization systems [25,26]. With the surging of smartphone sensing and wireless networking techniques, crowdsensing has become a promising paradigm for cross-space and large-scale sensing [27,28,29,30,31,32,33]. Gupta A, et al. used crowdsourcing methods to estimate road gradients from multiple sources or vehicles to improve the accuracy and robustness of the system [34]. Zahedi, et al. suggested a new estimation technique that implements multi-microphone noise reduction by enforcing a power constraint on the estimation problem [35]. Seid, et al. presented a mobile crowd-sensing-based road surface monitoring using smartphone sensors and a LoRaWAN network, enabling the generation of reports of road conditions and anomalies [36]. As technology expands in every industry, distributed technology [37] becomes more and more common. Qiu, et al. implemented a distributed diagnosis under bounded-delay communication of immediately forwarded local observations [38]. In summary, the literature studies on crowd-sensing have been applied to various life phenomena. Motivated by these works, we use dual-microphone smartphones as sensors to estimate the position of the thunderclaps using crowdsensing mechanisms.

## 3. A System Model and Definitions

Our ThunderLoc system transfers data from a dual-microphone smartphone to the cloud platform through a crowd-sensing mechanism. As far as we know, most smartphones are currently equipped with dual microphones, so our solution is feasible. ThunderLoc uses high sampling rate stereo-recording data to estimate the location of thunder. If thunder reaches the left microphone first, it is considered that the thunder may be located in the left area of the vertical bisector of the two microphones; otherwise, it may be located in the right area.

**Figure 2:**
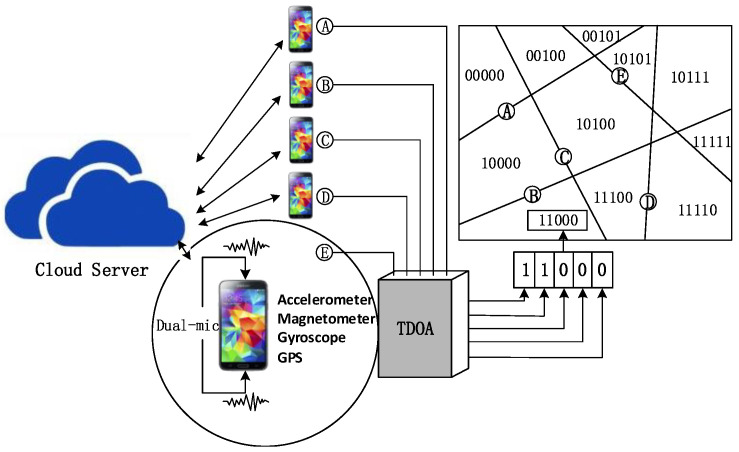
System overview.

Figure 2 shows the layout of the ThunderLoc system. This research uses a crowdsourcing mechanism to form a self-organizing sensor array from multiple dual-microphone smartphones. In order to simplify this problem, we considered a 2D localization space composed of *N* dual-microphone smartphones, and the signals acquired by the dual microphones of the same smartphone are synchronized. The vertical bisector of the *N* dual microphone divides the localization space into small sub-areas.

**Figure 3:**
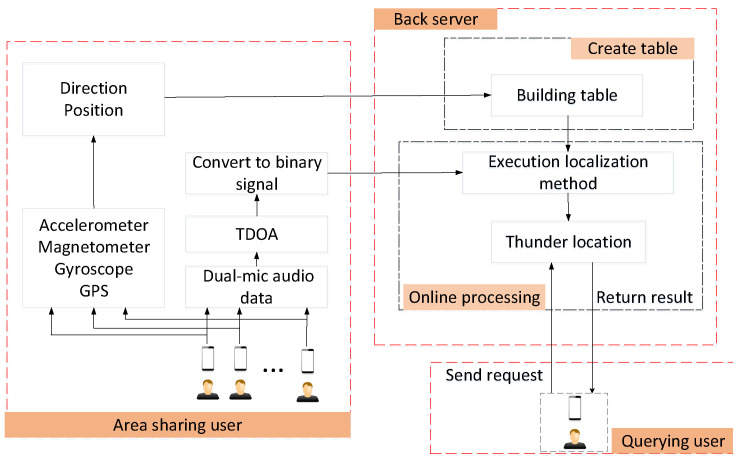
System architecture.

This paper uses map information and a measured binary sequence to solve the thunder-localization problem. In short, the operation of the ThunderLoc system is shown in Figure 3. In the client, the location information and direction information of the smartphone can be estimated through GPS and motion sensors (such as accelerometers and gyroscopes) integrated with the smartphone, respectively. When thunder occurs, each smartphone will detect the TDOA of the two synchronization signals recorded by the dual microphones and then obtain the binary left/right data according to the sign of the TDOA. The APP in each smartphone uploads the binary measurement data, the location information, and the direction information of the smartphone to the cloud-computing platform together. During the data collection period, participants may not even know what collection task they are involved in.

In addition, ThunderLoc encourages participants to manually upload sensing data to the cloud-computing platform when they hear thunder. The thunder-localization algorithm runs on the cloud-computing platform to locate the dominant thunder. Using these randomly distributed smartphones, the area can be divided into sub-areas according to the location and direction of the smartphone node. This will naturally give an *N* vector binary code called the binary detection sequence in the cloud-computing platform. As shown in Figure 2, it embeds the relative positional relationship between the smartphone node and thunder. Using pre-calculated sub-region division, the location of thunder can be estimated by processing the binary detection sequence.

In the next section, we will first provide the “basic” ThunderLoc system and then propose a “robust” ThunderLoc to solve the disadvantages in actual application scenarios.

## 4. Our Proposed Distributed Thunder Localization System

ThunderLoc used a client-server system architecture to allow data transfer from mobile devices to server stations or cloud platforms. When thunder occurs, the smartphone will be triggered to transmit its position, direction, and binary measurement data to the server. The server will then process the received data to achieve thunder localization. In this section, we first introduce the design of the client application and then introduce two localization methods on the server side in the next two subsections.

### 4.1. Data Collection on Client

**Figure 4:**
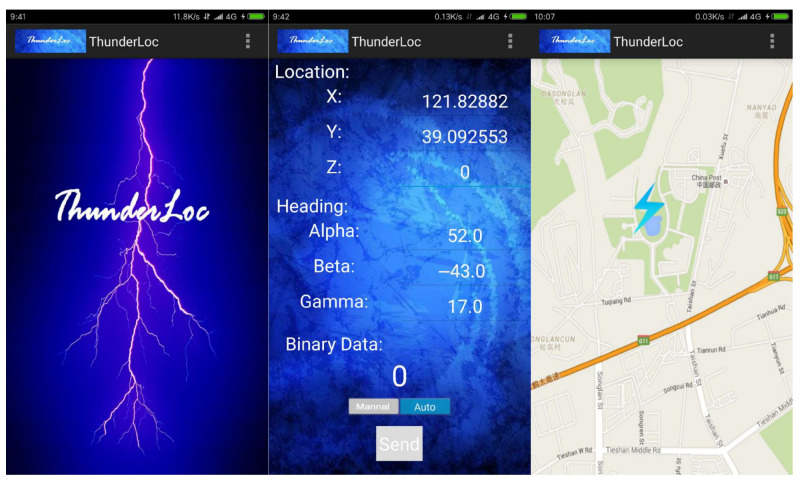
ThunderLoc application on smartphones.

**Figure 5:**
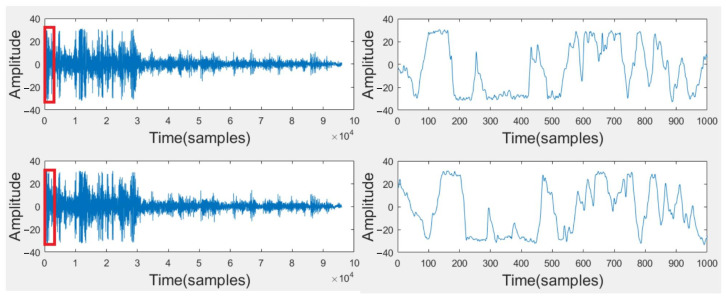
Dual-microphone acoustic signals.

Figure 4 shows a screenshot of the application and the visualization used by Raiden participants. Figure 5 shows the sound signals from the two channels of the dual microphones in the mobile phone. These signals are collected using remote sensing. Above is the acoustic signal collected by the top microphone, and below is the bottom. The arrival signals in the red boxes are amplified on the right. It is not difficult to see that the sound of thunder first reaches the top microphone.

**Figure 6:**
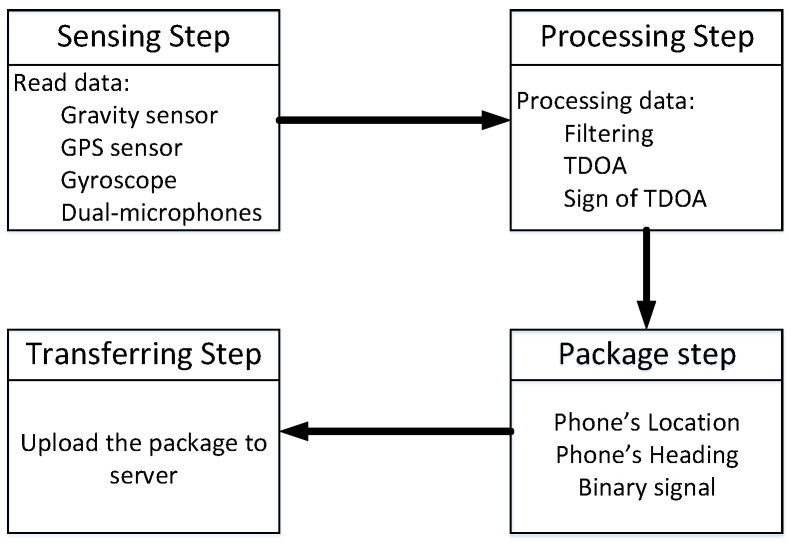
The diagram of signal processing of acoustic signals.

Figure 6 describes the flow of the ThunderLoc client application. The ThunderLoc client application has two main independent threads: the perception thread and the communication thread. The sensing thread handles the interaction with the main application and onboard sensors, and the communication thread handles local storage, the transmission of recorded sensor events, and event queues in case of poor network connection. Considering the energy issue, the continuous sending of data from the mobile device to the server is not an energy-saving solution. To solve this problem, the ThunderLoc client application uses a three-state model: idle mode, listening mode, and communication mode.

**Figure 7:**
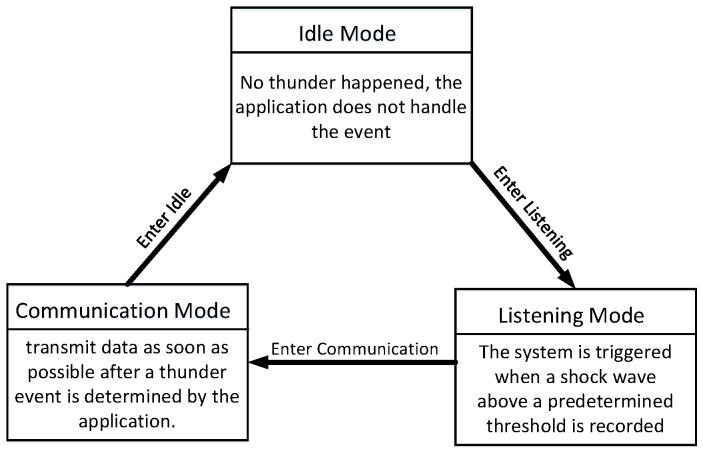
Modes of sensing of the client.

Figure 7 describes the different modes of client perception. When possible, thunder events are continuously recorded and sensed locally on the mobile phone; this model allows minimal data transmission to the server.

- Idle Mode: In order to determine the direction of the phone, the mobile device must be stationary for a period of time while recording sensor data. The movement of the device is characterized by changes in the accelerometer and gyroscope readings. If the cumulative amount of movement is below the threshold, the device will be verified as static and then jump into listening mode.
- Listening Mode: Thunder is a sound shockwave caused by sudden and intense heating of the air in the lightning tunnel. When a shockwave exceeding a predetermined threshold is recorded, the system will be triggered. When the average energy experienced by any one dual microphone increases by four times, the trigger will be triggered.
- Communication Mode: In order to reduce the total delay of the ThunderLoc system, data must be transmitted as soon as possible after a thunder event occurs. The application records sensor readings about the direction and position of the smartphone and then immediately transmits these data to the server along with binary measurement data estimated from the two-channel acoustic signals of the dual microphones. If the communication service is unavailable during the thunder event, it stores a copy of these records locally and put them in the queue to be sent when the communication service is available again.

In addition to its method of implicitly collecting data, ThunderLoc also allows users to explicitly enter the location, direction, and binary measurement data—using “human beings as sensors” [39].

### 4.2. Basic Localization on Server Side

**Figure 8:**
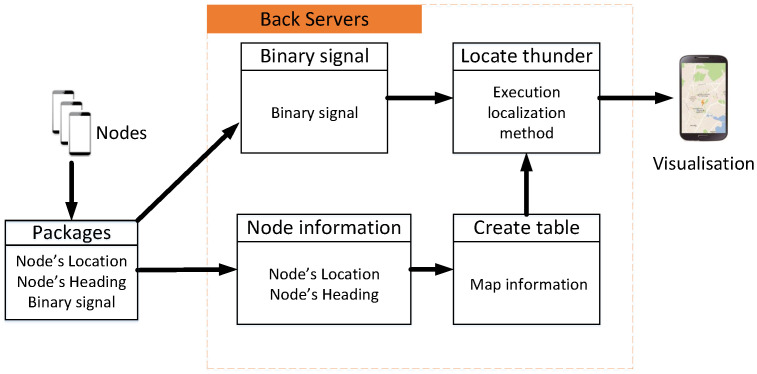
The diagram at cloud platform.

In this section, it is assumed that the location and direction of the smartphone are known and can be estimated using its GPS (or WiFi-based indoor localization) and motion sensors, respectively. Figure 8 describes the flow of the ThunderLoc server application. On the cloud-computing platform, the location and direction information of all smartphone nodes can be used to construct a spatial division map. Then, we can turn the sound source localization into a search problem in Hamming space.

First, we introduce the basic localization method. Let us consider randomly deploying a 4G network of *N* dual-microphone smartphones in a 2D area. The top-level idea of the basic ThunderLoc is to divide the entire localization area into certain sub-areas identified by the corresponding binary sequence. Given *N* smartphone nodes in the localization space, the total number of binary sequence combinations is theoretically $2^{N}$. However, in a real system, given that there are *N* smartphone nodes in the localization space, the number of possible combinations of binary sequences is only $(N^{2}+N+2)/2$. The lower dimension of the sequence table can correct errors in the measured sequence. These errors are nodes that incorrectly reverse the values of 0 and 1. This is one of the reasons why our proposed algorithm performs well under adverse conditions. With the increase in the number of smartphones, the localization system can achieve higher localization performance. Compared with dedicated microphone array hardware, this scalability is its unique advantage.

Binary sensor model: We propose a binary sensor model in which the TDOA of the two-channel sound signal is reliably converted into one bit of information: thunder is on the left or right of the vertical bisector of the two microphones. We use the symbol of TDOA as 1-bit measurement information, which shows left and right information about the direction of incoming thunder. The use of 1-bit measurement information enables inexpensive detection and minimal communication load. For each dual-microphone smartphone node, TDOA is calculated through traditional time delay estimation methods, so the binary sequence of thunder can be obtained.

**Figure 9:**
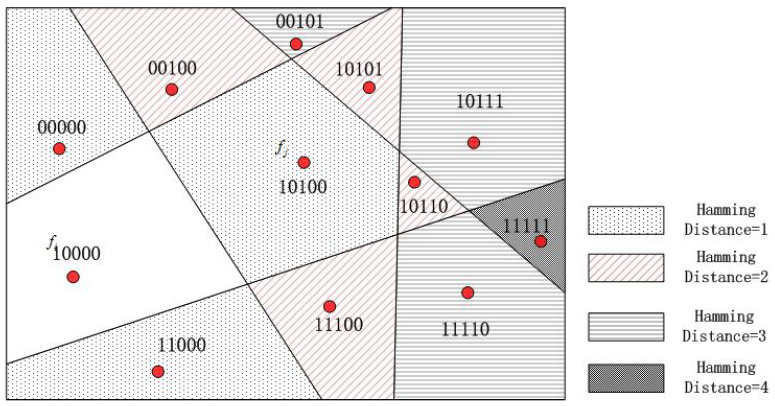
Hamming distance vs. geographic distance.

Hamming distance: For the two faces $f_{i}$ and $f_{j}$ in Figure 9, there are now two types of distance: (i) $f_{i}$ and $f_{j}$ geographic distance between the center points $GD(f_{i},f_{j})$, and (ii) Hamming distance $HD(f_{i},f_{j})$ [9]. Hamming distance is used to measure the dimensionality of two vectors with different values.

**Figure 10:**
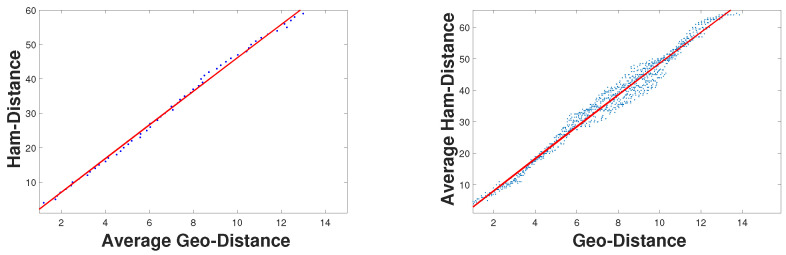
Relationship between Hamming distance and geographic distance.

From Figure 10, we can see that the closer the geographical distance between the two faces, the smaller the Hamming distance. In other words, geographic distance is positively related to Hamming distance. For these two distances, we have the following observations:

(1) $$GD\left(f_{i},f_{j}\right)\propto HD\left(f_{i},f_{j}\right).$$

Equation (1) indicates that the Hamming distance between two faces is roughly proportional to their geographic distance. This is because the longer the geographic distance, the better the chance of crossing the bisector, resulting in more flips. Therefore, when the Hamming distance is close to zero, the location is very close. In addition, we conducted a lot of simulation experiments to prove this point. The experimental results can be seen in Figure 10, and prove that the Hamming distance increases almost linearly with the increase in geographic distance. Given a binary query vector from multiple smartphones, we can estimate the location by retrieving the vector in a pre-database and finding the smallest Hamming distance. In other words, we can find the target location by querying the minimum Hamming distance.

The basic ThunderLoc consists of three steps:

(1) Divide the space into *p* grid points: Suppose that in $P{}_{i},i=1,\ldots ,p$, j−th smartphone $n{}_{j},j=1,\ldots ,N$ can calculate TDOA and get the symbol of TDOA. Then, we can get the binary code $C{}_{i}{,}_{j}\in \left\{0,1\right\}$ with the notation of TDOA. Combining the binary codes of all the *N* smartphones, we can obtain the *N* vector binary sequence $D{}_{i},i=1,\ldots ,p$. Obtain a database of discrete points about *p*, and the items in the database are $S_{i}=\left\{D_{i},P_{i}\right\}$.

(2) Calculate the binary sequence of the target *T*: When the sound source emits sound, all smartphone nodes will calculate TDOA, then obtain the symbol of TDOA and determine the binary code. The $TDOA_{i}$ of the *i*-th smartphone can be calculated using a delay estimation algorithm (such as GCC), and the binary code of the i−th smartphone can be defined by calculating the symbol of TDOA:

(2) $$Binary\_data_{i}=\left\{\begin{array}{c} 1,\;\;\mathrm{if}\;\;TDOA_{i}\geq 0;\\ 0,\;\;\mathrm{if}\;\;TDOA_{i}<0. \end{array}\right.$$

(3) Process the binary sequence *T*: Calculate the Hamming distance between *T* and each D(i), and find the sound source location by searching for the minimum value of the Hamming distance. The final location estimate is the average of all points with the smallest Hamming distance.

We can calculate the time complexity in three steps. In step 1, we set up a database, including calculating the TDOA of a pair of microphones and using only linear time to get its sign. For each grid point, we need to calculate the TDOA of *N* to the microphone, and there are *P* grid points, so the time complexity in step 1 is $O\left(PN\right)$. Similarly, the time complexity of step 2 is $O\left(N\right)$. In step 3, the time complexity of search processing is $O\left(PN\right)$. Therefore, the overall time complexity is $O\left(PN\right)$.

**Figure 11:**
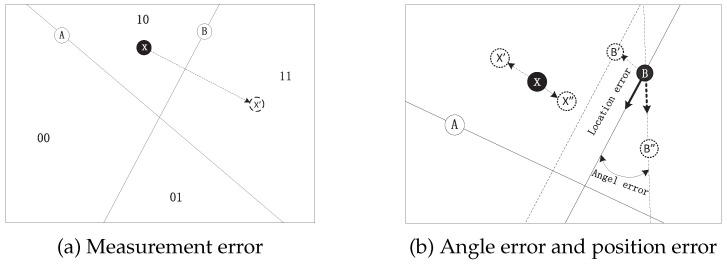
The impact of variety of errors.

The bit flip of the measurement data, the wrong location, and orientation parameters will affect the localization performance, as shown in Figure 11. As shown in Figure 11a, if the measured binary data from node B is flipped, the binary sequence “10” becomes “11,” which reduces the accuracy of the localization system. In Figure 11b, if the location measurement or angle measurement of sensor B is not accurate, there will be a certain distance between the actual location and the estimated location [9]. According to the above, the basic ThunderLoc is not suitable for practical applications. In the next section, we will propose a reliable localization solution for the measurement error, localization error, and direction error of the smartphone.

### 4.3. Robust Localization on Server Side

In practical applications, the bit-flip problem mentioned earlier should be considered. In addition, the measurement error and the uncertainty of node parameters will also affect the localization performance. In this section, a robust ThunderLoc is designed to avoid these problems. The results we obtained show that in practical applications, robust localization methods can greatly reduce localization errors.

In order to improve the robustness of the localization system, the possible coordinates of thunder can be calculated by choosing K minimum Hamming distances instead of the minimum Hamming distances. Then, the centroid estimation method is used to set the centroid of all possible points as the estimated location of thunder.

The Gaussian function is suitable for weighting discrete points according to their Hamming distance:

(3) $$w_{i}=exp\left(-HD\left(T,D_{i}\right)/N\sigma^{2}\right).$$

where σ is the parameter used to decide the weighting strategy.

Based on the normalized weight ${\bar{w}}_{i}$, the final estimated location of the thunder is estimated by computing the weighted sum of all grid points:

(4) $${\bar{w}}_{i}=\frac{w_{i}}{\sum_{i=1}^{P}w_{i}},$$

(5) $$\hat{x}=\sum_{i=1}^{P}{\bar{w}}_{i}\cdot p_{i}.$$

Compared with the basic ThunderLoc, the robust ThunderLoc uses all possible points to locate thunder. Although the amount of calculation has increased, the robust ThunderLoc has two advantages: (i) it avoids the problem of selecting the parameter *K*; (ii) it is more robust with respect to measurement errors and node parameter errors. Our evaluation in Section 5 shows that a robust localization method greatly enhances the robustness of the localization system.

We can calculate the time complexity of the robust ThunderLoc following three steps. From the algorithm, we can see that the time cost is the same as the basic localization method in steps 1 and 2. In addition, in step 3, we calculate the normalized weight of each point, and the corresponding time complexity is $O\left(P\right)$. In the last step, we calculate the location of thunder by the sum of the calculation of the Hamming distance. This is the core of the proposed ThunderLoc system, which can be effectively implemented through Boolean XOR operations on a cloud-computing platform with GPU and FPGA support. In the next section, the extensive experimental results demonstrate that ThunderLoc outperforms the main existing schemes in terms of effectively locating position and having good robustness.

## 5. Performance Analyisis

### 5.1. The Impact of Background Noises

Above, we proposed the basic ThunderLoc and the robust ThunderLoc to realize thunder localization by using a dual-microphone smartphone. The binary left/right measurement data of each smartphone comes from the TDOA symbol between two audio signals recorded with dual microphones. However, in an actual localization system, due to the influence of background noise in the binary left/right calculation phase of each smartphone, bit-flipping problems may occur, resulting in localization errors.

The lower SNR makes the measurement results from the smartphone more unreliable. We use the SNR of each smartphone node as a reliability index and then use the reliability information to calculate the Hamming distance.

The new localization method is the same as the robust localization method described in Algorithm 1, the only difference being that the weighted Hamming distance is used instead of the traditional Hamming distance:

(6) $$WHD=\sum_{i=1}^{N}F\left(i\right)\left(T\left(i\right)⊕D_{j}\left(i\right)\right).$$

**Algorithm 1:** Robust ThunderLoc
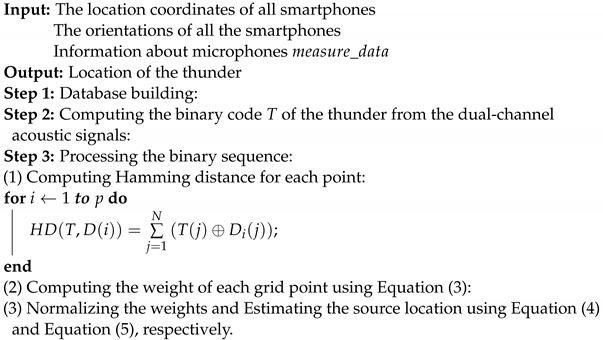


### 5.2. The Impact of Natural Environmental Noise

In the environment we live in, when thunder and lightning occur on a rainy day, it will also produce a lot of interference noises in the background. In this section, to locate thunder accurately, we need to distinguish between other disturbing noises in the environment and thunder. For the interference noise in the background, we train a neural network when different kinds of background noise happen at the same time, using machine learning methods to distinguish them. After a training phase, we can distinguish different background noises. The way we mainly use the model built by the TensorFlow machine learning framework. In the simulation, we use 20 different noise samples and thunder samples as backgrounds for training and then distinguish them.

**Figure 12:**
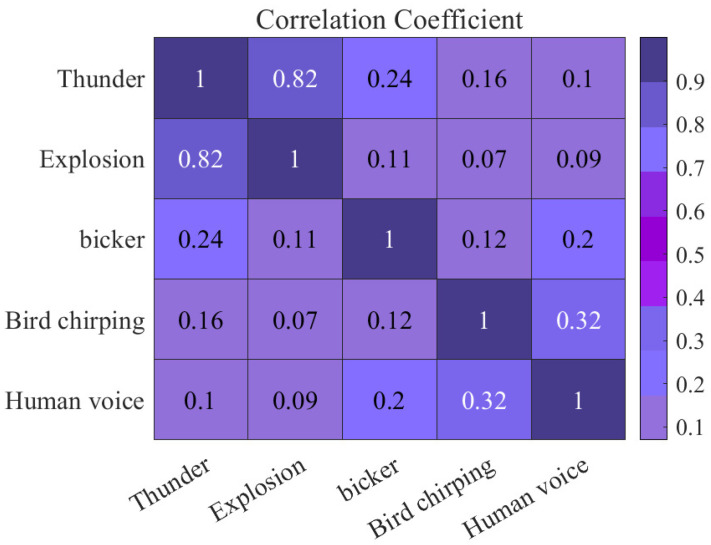
Interference of other noise in the environment.

The background noise of the correlation result of the experiment is shown in Figure 12. In comparison with the sound of rain, birds, and the human voice, we find that the noise of an explosion is more disturbing, and the accuracy is low in the same condition.

### 5.3. System Scalability and Multiple Thunder Localization

**Figure 13:**
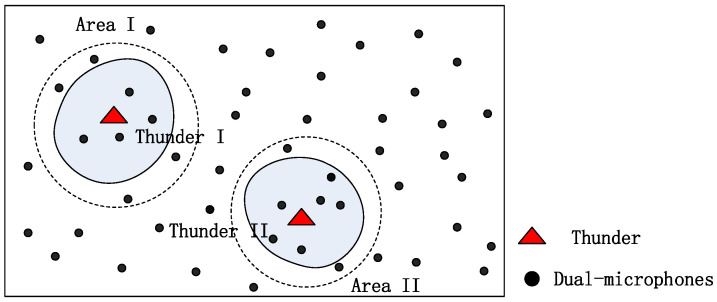
System scalability for large-scale systems.

Our system is evaluated in a small campus area but can be extended to larger areas. Figure 13 describes system scalability. In Figure 13, the signal from the thunder in a large-scale system can only cover a limited area, which means that when we measure a binary sequence, only a small number of microphones close to thunder are effective. Therefore, the binary sequence of thunder is shorter than N (there are N dual-microphone smartphones). We can use range R to place a large system in a smaller area that is large enough to cover the coverage of the thunder signal. The small area can be handled as the algorithm proposed in the previous part of this article. In this way, the length of the binary sequence is shorter, and the matching is more effective.

Locating multiple sound sources that are active at the same time is a more difficult task. In order to avoid the conflict of multiple sound sources, the localization of multiple sound sources must be able to uniquely identify each thunder signal, which is beyond the scope of this article, so we will not discuss it too much in this article. For example, if multiple claps of thunder are set, as shown in Figure 13, we can calculate the locations of the two mines because Thunder I is far enough from Thunder II. Smartphones near the effective area I can handle Thunder I, and other smartphones near the effective area II can handle Thunder II.

### 5.4. Time Synchronization and Energy Efficiency

In our system, we calculate the sign of TDOA by using two simultaneous audio signals from the dual microphones of each smartphone. There is no need for high-precision time synchronization between smartphones.

Energy efficiency is another issue with smartphone networks. We can activate the ThunderLoc application on the smartphone based on the thunderstorm warning in the weather forecast application. The ThunderLoc application will wake up only when it is raining.

### 5.5. 2D Simulation

In this section, we use MATLAB to evaluate our localization method in a 2D scene. In the simulation, we randomly distribute dual-microphone phones in an area of 10 km × 10 km, and the distribution is even; the distance between the two microphones is 14 cm, and the audio sampling rate is 48 kHz. In order to obtain a high degree of confidence, all statistics were run more than 3000 times and reported using the Root Mean Square Error (RMSE). The parameter σ in the powerful ThunderLoc has little effect on localization performance. By default, σ=1. We intend to conduct a comprehensive evaluation and comparison of the two proposed localization methods from different aspects, including the influence of the number of nodes, the number of faulty nodes, the influence of node localization errors, and the influence of angle errors. By default, there are 100 mobile phones and only one thunderclap. In order to simulate the influence of the location and orientation of the smartphone node on the localization accuracy, all simulations add a certain amount of location and angle errors. By default, the angle error range is 5 degrees, and the localization error range is 100 m. The results of the simulation evaluation are as follows.

**Figure 14:**
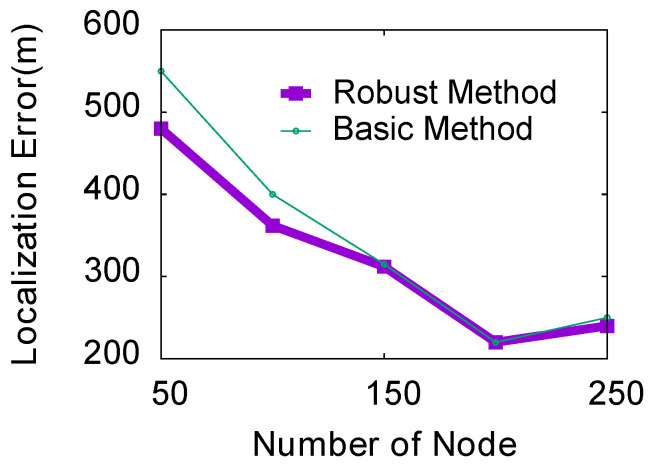
Impact of the number of nodes.

**(1) Impact of the number of nodes:** In this experiment, we use 10 as the step size to study the localization error when the number of nodes is between 50 and 250. We run the simulation with two samples of TDOA error, and other simulation parameters are set by default. As shown in Figure 14, as the number of mobile phones increases, the entire area can be divided into more grids, thereby achieving more accurate localization estimates for both localization methods. When the number of mobile phones is small, the performance of the robust ThunderLoc will be better than that of the basic ThunderLoc. As the number of participants increases, the two localization methods can achieve almost the same localization performance.

**Figure 15:**
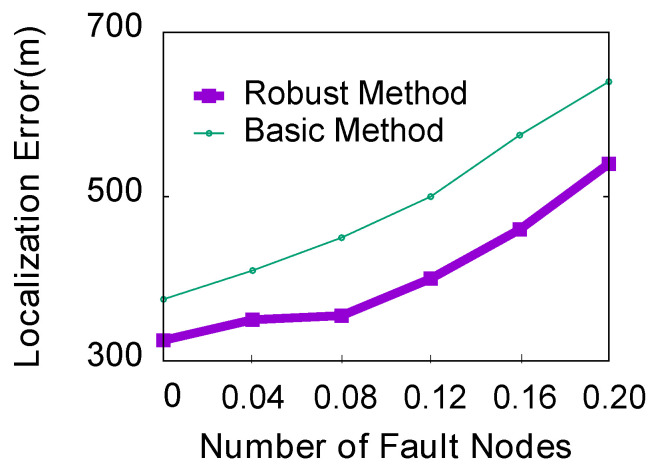
Impact of the number of error nodes.

**(2) Impact of the number of error nodes:** In practical applications, if the two microphones are close to each other along the direction of event propagation, they will detect the event almost simultaneously. In this case, the left/right binary data in the sequence may be reversed. We call this smartphone an “error node.” In this experiment, we try to compare the percentages of error nodes between the two methods, ranging from 0 to 0.2, with a step size of 0.04. Other simulation parameters are still the default settings. Figure 15 indicates that for both methods, the localization error increases with the increase in the number of faulty nodes. It also shows that as the number of error nodes increases, the average localization error of the robust ThunderLoc will be smaller than that of the basic ThunderLoc.

**Figure 16:**
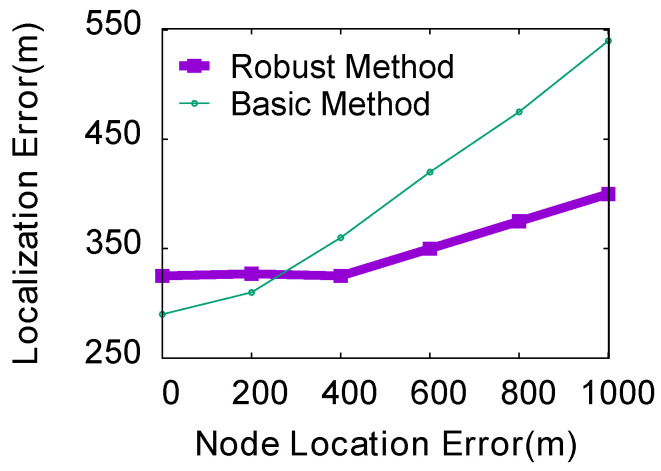
Impact of the localization error.

**(3) Impact of the localization error of nodes:** In the experiment, we evaluated the influence of the localization error of the basic ThunderLoc and the robust ThunderLoc nodes ranging from 0 to 1000 m in steps of 50 m. We keep the default values for other simulation parameters. Figure 16 shows that as the localization error of the smartphone node increases, the average localization error of the robust ThunderLoc is much smaller than that of the basic ThunderLoc.

**Figure 17:**
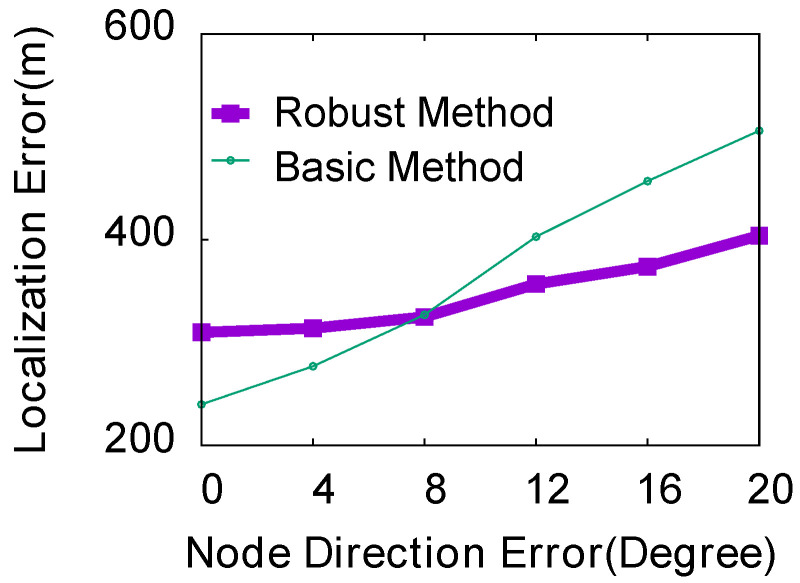
Impact of orientation error.

**(4) Impact of the orientation error of nodes:** In the experiment, we evaluated the influence of the angle error of the basic ThunderLoc and robust ThunderLoc nodes in the range of 0 to 20 degrees with a step size of 2. We keep the default values for other simulation parameters. Since there is an error in the direction of the node, we can guess that the error in the direction may affect the localization accuracy. As shown in Figure 17, the localization error graphs of the two methods increase as the angle error of the node increases. When the angle error of the node is relatively large, the robust average localization error ThunderLoc is much smaller than the basic ThunderLoc, and the robust localization accuracy ThunderLoc is much better than the basic ThunderLoc.

### 5.6. 3D Simulation

**Figure 18:**
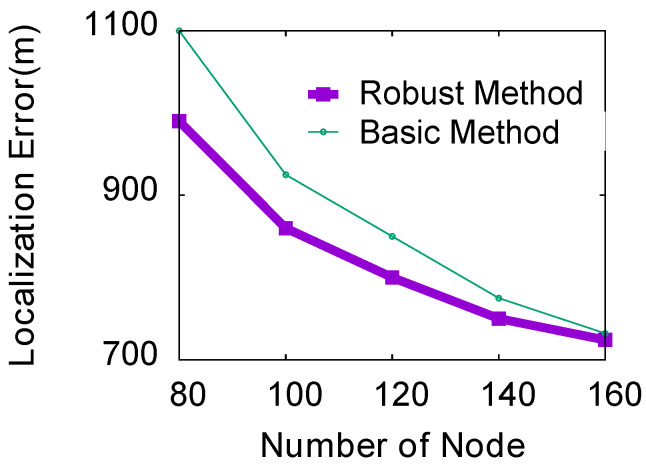
Thunder localization in 3D space.

The height of thunder should be considered in some applications, such as the reconstruction of lightning channels. In addition, if we do not consider the altitude, the sound of explosions on the ground will activate our system by mistake. In this section, we evaluate localization methods in 3D scenes. In the simulation, we randomly deployed 100 dual-microphone smartphones to cover an area of 10 km × 10 km. The height of the mobile phone is about 10 m, and the height of the thunder is about 3 km. The number of nodes is 100, and the angular error range of the smartphone is five. Due to space constraints, we only study the impact of the number of smartphones in the range of 80 to 160. The localization results of the experiment are shown in Figure 18. Compared with a 2D scene, the localization error of the same number of smartphones in 3D space is much larger. The reason is that the localization error is related to the size of the spatial grid divided by the vertical bisector of the dual microphones. In 3D scenes, more smartphones are needed to achieve high-precision localization.

After classifying the environmental noise through machine learning methods, we found that the interference of explosions had a greater impact on the localization of thunder. We conducted a simulation experiment of thunder and explosion in a 3D environment and evaluated the performance of the ThunderLoc system. Through investigations, we found that explosions generally occur in the range below 500 m in altitude and that thunder generally occurs in the range of 300–3000 m. We simulate the normal distribution of thunder in three dimensions at 300–3000 m and the explosion at 0–500 m as the real position and then make a prediction position within the error range for each real position. The ROC curve between the explosion and the thunder is used to analyze the robustness of the system.

**Figure 19:**
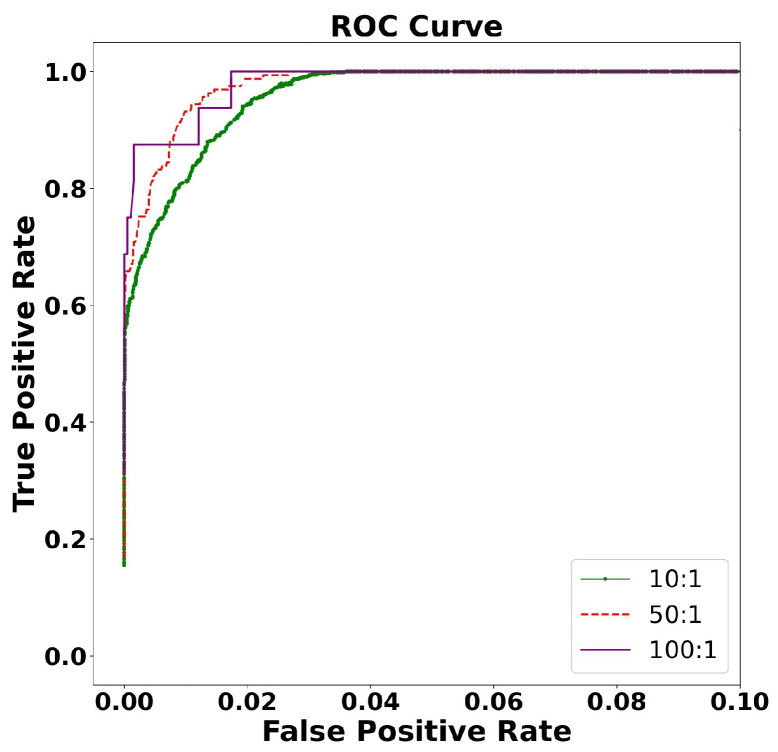
Different proportions of explosions and thunder of 3D ROC.

**Figure 20:**
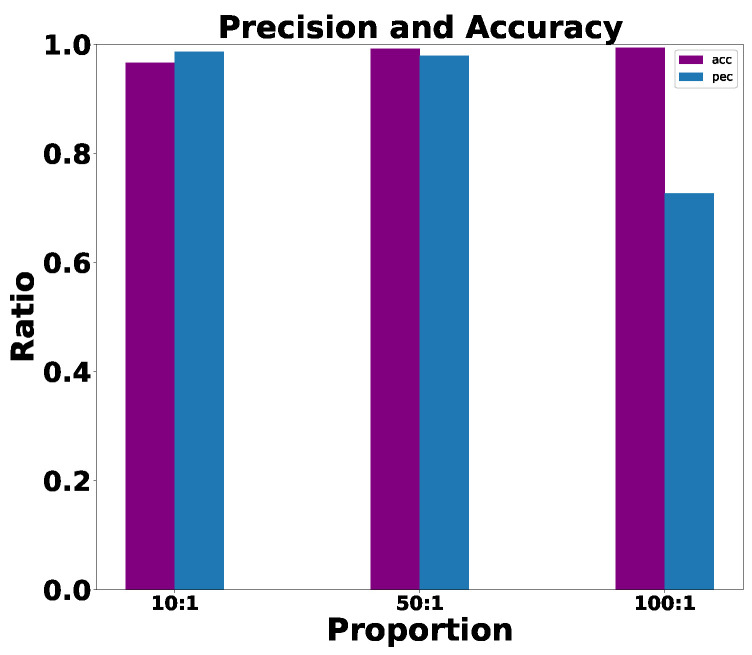
System in 3D precision and accuracy.

The ROC is shown in Figure 19. We found that the robustness of the system under different proportions of explosion and thunder is relatively stable and excellent. The precision and accuracy are shown in Figure 20, which, after analysis, are both above 80%, which shows good robustness.

### 5.7. Virtual Thunder Emulation

**Figure 21:**
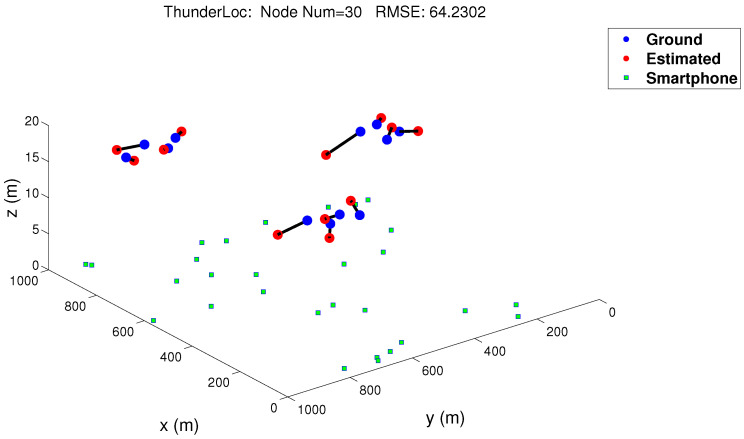
Experiment result.

The virtual thunder test is useful for correctly verifying the functionality of our ThunderLoc project. We use 30 dual-microphone smartphones as sensor nodes and connect them through a wireless router. The distance between the two microphones is 14 cm. We set 30 nodes in 1 km × 1 km space, and there was only one goal during the experiment. In order to simulate thunder, we broadcast the recorded thunder signal through speakers on the tops of three buildings. Smartphones are randomly deployed around buildings. The localization result is shown in Figure 21, where the blue square represents the dual-microphone smartphone, the blue circle and the red circle represent the ground truth, and the location estimated by a robust localization method, respectively [10]. The result is based on 30 nodes, and the root mean square error is 64.2302 m. The arrow goes from the estimated location of the virtual thunder to the actual location. As shown in Figure 21, the estimated location is close to the actual location, and the localization error is within the expected range, which means that our proposed ThunderLoc system can effectively locate thunder through mass perception.

## 6. Discussion

### 6.1. Robustness

In actual localization scenarios, common error sources mainly include these parts. The first is the position error of the node. Whether it is a large-scale GPS or a small-scale man-made measurement, the estimation of the node position will be biased. Then there is the direction and angle error of the mobile phone; factors such as gyroscope failure, magnetic field interference, etc., will cause large angle errors, which cannot be avoided. Then there is the deviation of the measured data, of which the most serious is the bit flip, that is, the mobile phone judging that the sound source is inverted on the left or right of the vertical line of the dual microphones. When the sound source is near the vertical line of the dual microphones, it is easy to occur. Bit flipping is usually rare. Once bit flipping occurs, it will have a greater impact on localization. Finally, a node fails, or data fails to be uploaded to the server in time, which is also a common source of error in wireless sensor networks.

In view of the above-mentioned types of errors, our system has a certain fault tolerance, which is reflected in the following aspects. First, for the problem of measurement data error, we designed a TDOA screening strategy in the algorithm to make full use of the potential of each part of the signal to judge the accuracy of TDOA and realize the selection, quantification, or removal of TDOA. It not only guarantees the accuracy of the measurement data but also retains part of the TDOA error information. Second, for node position error, angle error, and bit flipping, the localization algorithm proposed in this paper takes these situations into account and proposes a corresponding fault tolerance mechanism. In a large-scale localization scenario, the node only needs to use the TDOA symbol; even if the bit flip occurs, the system can still find the target area where the sound source is located, In small-range localization, the combination of space division and TDOA can further improve the accuracy. Third, there are certain advantages to the number of nodes. Smartphones have been widely used around the world, and most mobile phones are equipped with dual microphones. If the system can be promoted, it is equivalent to deploying nodes in various places. A node failure or measurement data error can be compensated by the measurement data of other nodes to obtain an accurate sound source location.

### 6.2. Overhead

The localization system designed in this paper also has certain advantages in terms of overhead. Common sound source localization algorithms, such as common TDOA hyperbolic intersection localization, etc., often require strict clock synchronization between sensor nodes. This paper uses a dual-microphone mobile phone as a sensor to calculate the time difference TDOA of the sound signal reaching the two microphones of the same mobile phone. Since the two microphones are integrated with the same device, they are inherently synchronized and do not require clock synchronization between nodes, which reduces the amount of additional data traffic.

In the wireless sensor network, continuous communication and interaction between nodes or between nodes and servers are required, and the data transmission volume of each communication is also an important factor affecting system performance. An important idea of this article is to quantify the measurement data TDOA, determine whether the sound source is on the left or right side of the vertical line in the dual microphones, divide the localization space, and generate one-bit measurement data. The larger the number of mobile phones, the smaller the area of the sub-region where the sound source is located. When there are enough mobile phones, this area can even be used to represent the location of the sound source. At this time, only one bit of information is transmitted between communication devices, which greatly reduces the amount of communication data. At the same time, the system allocates signal collection, processing, and TDOA calculations to each mobile phone node, reducing the burden on the server to process data.

In terms of development costs, the system uses smartphones that are popular in society as sensor nodes to fully develop the application potential of sensors in intelligence, eliminating the need to purchase dedicated microphone arrays and other sensor equipment and saving system development costs.

### 6.3. Engineering Applications

We apply this localization scheme to forest wildfire-prediction work to verify the robustness and practicability of the system. Lightning is one of the causes of forest wildfires. Wildfires are natural disasters with severe consequences that are worryingly worsening for many lightning and climate-affected areas of our planet. We capture thunder information to locate lightning. By capturing and analyzing lightning information in a very short time, we are able to quickly locate the position of lightning strikes. Thus, we can predict the extent of wildfires in time and take effective measures to control their spread.

**Figure 22:**
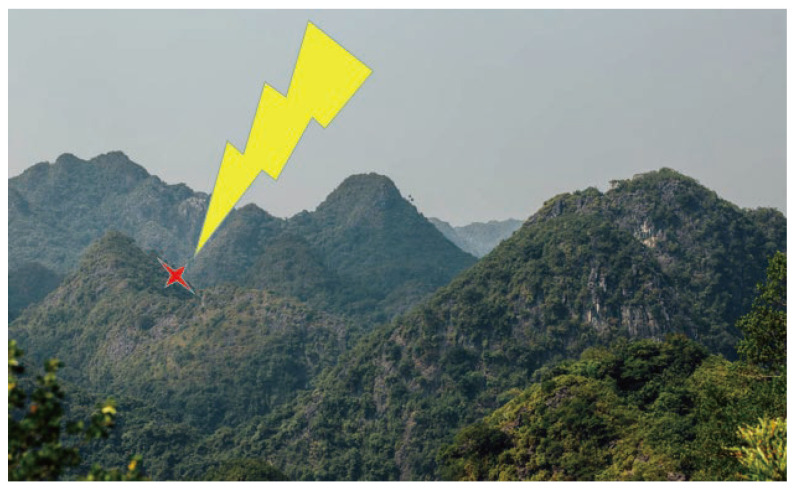
Forest Fire Prediction Using Thunder.

As shown in Figure 22, the yellow icon represents lightning, and the red icon represents the location of the wildfire, and our proposed system can accurately and timely locate the location of the wildfire. Using 100 mobile smart devices, the thunder-localization system can process the collected information and generate the longitude and latitude coordinates of the lightning strike point. We can easily observe the location of the lightning strike point on the mobile phone app. For continuous thunder, the system can accurately locate different lightning locations. The localization error of the thunder is within a controllable range. A series of experiments show that the system can accurately predict the location of possible wildfires. The actual test proves that our proposed system has strong practicability and accuracy.

## 7. Conclusions

In this paper, we designed ThunderLoc, a novel thunder-localization system that uses the common IoT devices in dual-microphone smartphones to achieve thunder localization using unreliable binary node sequences through crowdsensing mechanisms. The proposed design uses binary sequences from a dual-microphone smartphone to express thunder localization as a search problem in Hamming space. Since our ThunderLoc system runs on COTS smartphones and supports spontaneous installation, it is possible to enable various thunder-localization systems. In addition to the basic design, a robust localization method is also proposed to improve localization performance in practical applications. Our system has been verified and evaluated through analysis, extensive simulation, and test bench experiments. The test results show that this method can effectively use the crowdsensing mechanism to achieve thunder localization. The extensive experimental results demonstrate that ThunderLoc outperforms the main existing schemes in terms of good robustness and the effective location of position. Our next step is to study incentive mechanisms to increase the participation rate of the people.

## Data Availability

The data presented in this study are available on request from the corresponding author.
